# Emerging Infections Due to *Shewanella* spp.: A Case Series of 128 Cases Over 10 Years

**DOI:** 10.3389/fmed.2022.850938

**Published:** 2022-04-29

**Authors:** Wincy Wing-Sze Ng, Hoi-Ping Shum, Kelvin Kai-Wang To, Siddharth Sridhar

**Affiliations:** ^1^Department of Intensive Care, Pamela Youde Nethersole Eastern Hospital, Hong Kong, Hong Kong SAR, China; ^2^Department of Microbiology, School of Clinical Medicine, Li Ka Shing Faculty of Medicine, The University of Hong Kong, Hong Kong, Hong Kong SAR, China

**Keywords:** *Shewanella* infection, *Shewanella algae*, *Shewanella putrefaciens*, *Shewanella* species, gram negative bacilli

## Abstract

**Background:**

*Shewanella* species are emerging pathogens that can cause severe hepatobiliary, skin and soft tissue, gastrointestinal, respiratory infections, and bacteremia. Here we reported the largest case series of infections caused by *Shewanella* species.

**Aim:**

To identify the clinical features and risk factors predisposing to *Shewanella* infections. To evaluate resistance pattern of *Shewanella* species and appropriateness of antibiotic use in the study cohort.

**Methods:**

Patients admitted to a regional hospital in Hong Kong with *Shewanella* species infection from April 1, 2010 to December 31, 2020 were included. Demographics, antibiotics, microbiology, and outcomes were retrospectively analyzed.

**Findings:**

Over the 10 years, we identified 128 patients with *Shewanella* species infection. 61.7% were male with a median age of 78 (IQR 65–87). Important underlying diseases included hepatobiliary diseases (63.3%), malignancy (26.6%), chronic kidney disease or end-stage renal failure (25.8%), and diabetes mellitus (22.7%). Hepatobiliary infections (60.4%) were the most common clinical manifestation. Majority (92.2%) were infected with *Shewanella algae*, while 7.8% were infected with *Shewanella putrefaciens*. The identified organisms were usually susceptible to ceftazidime (98.7%), gentamicin (97.4%), cefoperazone-sulbactam (93.5%) and ciprofloxacin (90.3%). Imipenem-susceptible strains were only present in 76.6% of isolates.

**Conclusion:**

This largest case series suggested that *Shewanella* infections are commonly associated with underlying comorbidities, especially with hepatobiliary diseases and malignancy. Although *Shewanella species* remained largely susceptible to third and fourth generation cephalosporins and aminoglycosides, carbapenem resistance has been on a significant rise.

## Introduction

*Shewanella* species are Gram-negative bacilli that are motile and non-fermenting. It was first discovered 90 years ago by Derby and Hammer in 1931, during an investigation for the cause of surface taint on properly pasteurized butter. Contamination of dairy products with this organism led to despicable flavor and odor despite appropriate storage. The organism was initially given the name *Achromobacter putrefaciens*. Later in 1941, Long and Hammer proposed that it should be renamed as *Pseudomonas putrefaciens* under the genus of *Pseudomonas*. They also further isolated the organism from other dairy products like milk and cream, and other sources like soil, water samples, creamery floors, equipment and sewers. In subsequent decades, this species was implicated in spoilage of fish and fishery products by James Shewan and other authors (1). In 1985, this organism was reclassified to the family of *Vibrionaceae* as a result of phylogenetic studies. A new genus, *Shewanella*, was given to this organism in honor of James Shewan for his dedication to marine microbiology. Since its discovery 90 years ago, numerous case reports and case series have been published regarding this species. However, infections by *Shewanella* species were rare, and knowledge regarding *Shewanella*-associated infections remained limited.

## Materials and Methods

We conducted a retrospective analysis of patients with *Shewanella* infection over 10 years, from April 1, 2010 to December 31, 2020 in Pamela Youde Nethersole Eastern Hospital (PYNEH), a 1700-bed regional hospital in Hong Kong. Patients who had insufficient data for analysis were excluded. Data from the Clinical Management System, the Clinical Information System (IntelliVue Clinical Information Portfolio, Philips Medical, Amsterdam, Netherlands) and the medical records were reviewed retrospectively. Background medical comorbidities, demographics, clinical and microbiologic characteristics of *Shewanella* infection were retrieved and evaluated.

### Definitions

Clinical significance of *Shewanella* infections was classified into “definite,” “probable,” and “colonization” as described previously (2). “Definite” *Shewanella* infection was defined as isolation of *Shewanella* from a normally sterile site or pure growth from other infected sites as a single organism. “Probable” *Shewanella* infection was defined as isolation of *Shewanella* from infected sites, which is polymicrobial. “Colonization” by *Shewanella* was defined by the isolation of *Shewanella* from sites without clinical signs or symptoms of infection. Sites of isolation were classified into intra-abdominal specimens (including bile and peritoneal swabs), skin and soft tissue specimens, blood, respiratory tract, and other specimens.

Empirical antibiotics were defined as the antibiotics administered within 24 h of collection of specimens with *Shewanella* being isolated. Subsequent antibiotics were defined as the antibiotics administered beyond 24 h of specimen collection.

Clinical outcomes being evaluated included hospital length of stay, need for Intensive Care Unit (ICU) admission and hospital mortality. The Acute Physiology and Chronic Health Evaluation (APACHE) IV score was used in those admitted to the ICU to quantify disease severity and predict the risk of mortality in the index admission. It comprises 129 variables derived from the worst values in the initial 24 h of admission. Required variables for calculating the APACHE IV score include the patient's demographic and chronic health variables, physiological and laboratory parameters, and disease-specific subgroups.

### Microbiology

Clinical samples were submitted to the microbiology laboratory in PYNEH for incubation and processing. *Shewanella* species was identified by Matrix-Assisted Laser Desorption Ionization–Time-Of-Flight (MALDI-TOF) Mass Spectrometry (MS). Susceptibility testing was performed on the isolates to classify them into Resistant (R), Intermediate resistant (I) or Susceptible (S) strains to the tested antibiotics, based on the Clinical and Laboratory Standards Institute (CLSI) criteria. Before June 2018, the disc diffusion method was employed and the CLSI minimal inhibitory concentration (MIC) breakpoint for *Pseudomonas aeruginosa* was adopted. Since June 2018, the broth micro-dilution method was performed for susceptibility testing and the CLSI MIC breakpoint for Other Non-Enterobacteriaceae (ONE) was used.

### Ethics

This study was approved by the Hong Kong East Cluster Ethics Committee of the Hospital Authority (HKECREC-2020-072).

## Results

### Demographics and Medical Comorbidities

A total of 128 patients with *Shewanella* infection were identified during the 10-year study period (Table 1). 79 (61.7%) were men, and 49 (38.3%) were women. The median age was 78 (interquartile range, IQR 65–87). 81 patients (63.3%) had an underlying hepatobiliary disease, 34 (26.6%) had an underlying malignancy, 33 (25.8%) had chronic kidney disease or end-stage renal failure, and 29 (22.7%) had diabetes mellitus. Among the 34 patients with an underlying malignancy, 58.8% (*n* = 20) had cancer of the hepatobiliary tract.

**Table 1:** Clinical characteristics of Shewanella-associated sepsis.

Clinical characteristics (***n*** = 128)	
Gender	Male (*n* = 79)
	Female (*n* = 49)
	M:F ratio (1.6:1)
Age	78 (65–87)
Underlying conditions	Hepatobiliary disease (*n* = 81, 63.3%)
	Malignancy (*n* = 34, 26.6%)
	Hepatobiliary cancer (*n* = 20)
	Cholangiocarcinoma (*n* = 8)
	Pancreatic cancer (*n* = 6)
	Cancer of ampulla of Vater (*n* = 2)
	Cancer of gallbladder (*n* = 4)
	Colorectal cancer (n = 7)
	Gastric cancer (*n* = 2)
	Breast cancer (*n* = 2)
	Lymphoma (*n* = 2)
	Malignant melanoma (*n* = 2)
	Chronic kidney disease/End-stage renal failure (*n* = 33, 25.8%)
	Diabetes mellitus (*n* = 29, 22.7%)
Seawater exposure (*n* = 6, 4.7%)	
Mortality and morbidity	Hospital death (*n* = 11, 8.6%)
	Require ICU care (*n* = 11, 8.6%)
	ICU Length of stay (4, IQR 2-8)
	ICU mortality (*n* = 1, 9.1%)
	Hospital mortality (*n* = 2, 18.2%)
	APACHE IV risk of death 0.34 (IQR 0.15–0.77)
Clinical manifestations (specimen *n* = 154)	
Hepatobiliary infections (*n* = 93, 60.4%)	
	Gallstone or bile duct stone disease (*n* = 85)
Skin and soft tissue infections (*n* = 36, 23.4%)	
	Chronic wound (DM, chronic venous insufficiency, malignant ulcer) (*n* = 13)
Respiratory infections (*n* = 13, 8.4%)	
Gastrointestinal infections (*n* = 7, 4.5%)	
Others (*n* = 5, 3.2%)	

### Microbiology

Among 128 patients, 154 specimens were available for further analysis. Twenty-five episodes were considered “definite” infections, 75 episodes were considered “probable” infections, and 54 episodes were considered “colonization.” Co-isolates were very common (88.3%). The 3 most common organisms involved in co-infection with *Shewanella* were *Enterococcus* (49.4%), *Escherichia Coli* (48.1%) and *Klebsiella* species (27.3%). Table 2 listed all co-isolates involved in co-infection with *Shewanella*.

**Table 2:** Common organisms involved in co-infection with *Shewanella* (specimen *n* = 154).

No co-infection (*n* = 18, 11.6%)
With co-infection (*n* = 136, 88.3%)
Enterococcus (*n* = 76, 49.4%)
Escherichia Coli (*n* = 74, 48.1%)
Klebsiella (*n* = 42, 27.3%)
Streptococcus (*n* = 26, 16.9%)
Proteus (*n* = 25, 16.2%)
Bacteroides (*n* = 21, 13.6%)
Clostridium (*n* = 20, 13%)
Staphylococcus (*n* = 14, 9.1%)
Citrobacter (*n* = 13, 8.4%)
Enterobacter (*n* = 12, 7.8%)
Pseudomonas (*n* = 9, 5.8%)
Morganella (*n* = 8, 5.2%)
Aeromonas (*n* = 7, 4.5%)
Candida (*n* = 6, 3.9%)
Acinetobacter (*n* = 5, 3.2%)
Vibrio (*n* = 2, 1.3%)

*Shewanella* was isolated from intra-abdominal specimens (58.4%), skin and soft tissue specimens (20.1%), blood (8.4%), sputum (8.4%) and other specimens (4.7%). The most common clinical manifestation was hepatobiliary infections (*n* = 93, 60.4%), followed by skin and soft tissue (*n* = 36, 23.4%), respiratory (*n* = 13, 8.4%) and gastrointestinal (*n* = 7, 4.5%) infections. For those with hepatobiliary infections, 91.4% was related to gallstone or bile duct stone. For those with skin and soft tissue infection, 36.1% had chronic wounds due to either diabetes mellitus or chronic venous insufficiency. Seawater exposure was noted in 6 patients (4.7%), which were all classified as probable infections due to polymicrobial growth from infected sites.

Among 128 recruited patients, 118 patients (92.2%) were infected with *Shewanella algae*, while 10 patients (7.8%) were infected with *Shewanella putrefaciens*. The antibiotic susceptibility of *Shewanella* is shown in Table 3. The identified organisms were usually susceptible to ceftazidime (98.7%), gentamicin (97.4%), cefoperazone-sulbactam (93.5%), ciprofloxacin (90.3%), and piperacillin (88.3%). Only 76.6% of isolates were susceptible to imipenem-cilastatin. One isolate showed resistance to multiple antibiotics, including ceftazidime, cefoperazone-sulbactam, ciprofloxacin, piperacillin and imipenem-cilastatin, and remained susceptible only to gentamicin and ticarcillin-clavulanate.

**Table 3:** Antibiotic susceptibility of *Shewanella* (specimen *n* =154).

Ceftazidime (*n* = 152, 98.7%)
Gentamicin (*n* = 150, 97.4%)
Cefoperazone-sulbactam (*n* = 144, 93.5%)
Ciprofloxacin (*n* = 139, 90.3%)
Piperacillin (*n* = 136, 88.3%)
Imipenem-cilastatin (*n* = 118, 76.6%)

Amoxicillin-clavulanate was prescribed as an empirical antibiotic in 40.3% of the cases. The carbapenem group (ertapenem or meropenem) was chosen in 23.3% of the cases. Cefuroxime (8.4%), piperacillin-tazobactam (8.4%), aminoglycosides (gentamicin or amikacin, 8.4%), levofloxacin (7.1%) and ampicillin (3.2%) were less commonly used as empirical options. For subsequent antibiotics given after susceptibility results were available, carbapenem group (ertapenem, meropenem or imipenem-cilastatin, 28.6%) and piperacillin or piperacillin-tazobactam (23.3%) were commonly used. Amoxicillin-clavulanate (19.5%) became less prevalent than it was as an empirical choice.

Carbapenem group was the second most commonly prescribed empirical antibiotic class. Thirty out of 128 patients in our cohort received carbapenem empirically. Imipenem-resistant strains were detected in 5 of them with 1 hospital death, making up mortality rate of 20% (1 out of 5) among patients receiving carbapenems inappropriately. Out of the other 25 patients who received initial carbapenems appropriately, there was 1 hospital death resulting in mortality rate of 4% (1 out of 25). The two-tailed *P* value by Fisher's exact test was 0.3103, indicating that the association between presence of carbapenem resistance and mortality was not statistically significant.

### Clinical Outcomes

The median hospital length of stay was 5 days (IQR 2–12). The overall hospital mortality was 8.6%. Eleven patients (8.6%) required admission to the Intensive Care Unit (ICU). The median ICU length of stay was 4 days (IQR 2–8). The APACHE IV predicted risk of death was 0.34 (IQR 0.15–0.77). Among the patients admitted to the ICU, 1 patient (9.1%) died during the ICU stay, and 2 patients (18.2%) died during the hospitalization.

## Discussion

Since its discovery 90 years ago, there has been increasing understanding of the emerging *Shewanella* species. Nevertheless, knowledge regarding the clinical manifestations of *Shewanella* species is still primarily derived from isolated case reports or small case series. Here, we report the largest case series of *Shewanella*-associated infections in 128 patients over 10 years.

### Risk Factors

Male predominance was frequently described in literature (2–4). Consistent with the previous studies (2, 4, 5), the male to female ratio in our study was 1.6:1. It was postulated that male predominance could be a genetic predisposition or a result of greater occupational or recreational marine exposure (4, 5). However, marine exposure was only identified in 6 patients (4.7%) in our case series and cannot solely account for the male predominance.

Important underlying conditions associated with *Shewanella*-associated infections found in our study were hepatobiliary diseases (63.3%), malignancy (26.6%), chronic kidney disease (25.8%), and diabetes mellitus (22.7%). Hepatobiliary disease is well known to be associated with *Shewanella* infections in several case reports and case series (2, 3, 6–9). Chen et al. (3) reported a case series of 16 *Shewanella putrefaciens*-associated skin and soft tissue infections, in which hepatobiliary disorder has been identified in 75% of the cases. To et al. (2) reported half of the patients with *Shewanella* bacteremia had an underlying hepatobiliary disease, while Liu et al. (9) reported 28 out of 59 (47.4%) bacteremic patients having an underlying hepatobiliary disease. In our series, 81 out of 128 patients (63.3%) had an underlying hepatobiliary disease, which included choledocholithiasis, cholecystitis, cholangitis, liver abscess and malignant biliary obstruction. Eleven of our patients had *Shewanella* bacteremia, of which 6 (54.5% of bacteremic patients) had a hepatobiliary disease. One proposed mechanism is the lipophilic property and bile affinity of *Shewanella species*, as demonstrated by frequent isolation of the species from oil emulsions and fatty foods (3, 10). Another proposed mechanism was hepatic dysfunction resulting in iron overload, which suppresses neutrophilic activity and causes impaired phagocytosis, facilitating bacterial invasion and bacteremia (9, 11). This was speculated to be associated with *Shewanella* infections in expert opinion (2, 9).

Previous studies have also shown important associations between an underlying malignancy and *Shewanella* infections (2, 12, 13). Malignancy was present in 26.6% of patients in our study, and more than half (20 out of 34, 58.8%) were malignancies involving the hepatobiliary tract. Previous case reports postulated that cancer patients were predisposed to *Shewanella* infections due to immunosuppression (13), neutropenia (14, 15), and aggressive chemotherapeutic regimens being used (15).

Patients with chronic kidney disease (CKD) made up 25.8% of our study cohort. Significant correlation between CKD and *Shewanella* infection was not demonstrated in the existing literature. Isolated case reports have described peritonitis caused by *Shewanella* species in uremic patients on peritoneal dialysis (16–20). *Shewanella* infection (21) and septicemia (22) was also reported as rare case reports in patients receiving hemodialysis. These infections in hemodialysis patients reported in the literature were complications of the vascular access, either as catheter-related bloodstream infections or as an infected arterio-venous graft. In contrast to previous studies, none of our uremic patients suffered from *Shewanella* peritonitis due to underlying peritoneal dialysis. There were 3 cases of bacteremia in our subgroup of patients with CKD. None of these bacteremic patients had underlying vascular access. Two of them suffered from cholangitis, and 1 of them had necrotizing fasciitis. Our study was the first to demonstrate an association between *Shewanella*-associated infections and CKD *per se* and not as a complication of the dialysis modality employed. One possible explanation was the dysregulated iron homeostasis contributing to anemia in CKD, recently demonstrated to be related to raised hepcidin levels in uraemia (23). An overall positive iron balance may result (23). Iron then binds in high affinity to siderophores being produced by *Shewanella* species (24, 25).

A substantial portion of our cohort had underlying diabetes mellitus, accounting for approximately one-fifth (22.7%) of the cases. The association between diabetes and *Shewanella* infections has already been depicted in previous case reports and case series (2, 18, 20). Diabetic patients also frequently develop lower extremity ulcers. Patients with chronic wounds and ulcers are prone to skin and soft tissue infections by *Shewanella* species (26–33). These infections may or may not be preceded by contaminated water exposure to the wounds or ulcers. These infected skin lesions can potentially cause bacteremia, as described in one case series of 6 patients with an underlying burn wound or ulcerative skin lesion who developed *Shewanella putrefaciens* bacteremia (14). In our study, 13 patients had chronic wound conditions, including diabetics ulcers, chronic venous insufficiencies, and malignant melanoma with fungating wounds, making up 36.1% (13 out of 36) of all skin and soft tissue infections. Consistent with other studies (27), the majority of our patients with chronic wounds did not have preceding seawater exposure. The only case documented to have contaminated water exposure was a patient with chronic venous insufficiency who did dressing to his ulcer at home with non-sterile tap water (Case 6, Table 4).

**Table 4:** Cases with marine exposure.

**Case No**.	**Sex/Age**	**Underlying disease**	**Primary diagnosis**	**Relevant marine exposure history**	**Culture specimen**	**Clinical significance**
1	F/58	None	Pneumonia	Non-fatal drowning	Endotracheal aspirate	Probable
2	M/71	None	Traumatic amputation of thumb	Fisherman, thumb laceration by ropes while fishing	Wound swab	Probable
3	M/59	None	Post-traumatic wound infection	Fish farm owner, fall with elbow contusion	Wound swab	Probable
4	M/87	Diabetes mellitus	Scald wound infection	Recent pipeline damage causing home flooding, poor home hygiene Scald injury by hot water	Wound swab	Probable
5	M/75	None	Infected lower limb ulcer	Injury by broken furniture during typhoon with sea water contacted	Wound swab	Probable
6	M/57	None	Infected venous ulcer	Self-dressing to venous ulcer with non-sterile tap water	Wound swab	Probable

*Shewanella* species is a renowned marine organism, making seawater contact is a well-recognized risk factor for *Shewanella*-associated infections. Marine-related occupations or recreational activities such as diving (34), swimming (18), fishing (2, 18, 21) and crabbing (2) were identified to be important predisposing events to *Shewanella* infections. However, the importance of marine exposure in *Shewanella* infections varies in the literature. Holt et al. (34) reported a striking identification of seawater contact history in 85% (47 out of 55) cases of *Shewanella algae* ear infections. On the contrary, To et al. (2) identified only 2 out of 29 cases with a marine association, suggesting that an alternative vehicle or route of *Shewanella* infection may be present. In our series, only 6 out of 128 patients had documented seawater contact. All these cases had co-isolates present in the cultured specimens and were classified as “probable” infections. Most of them (5 out of 6) had skin and soft tissue infection after marine exposure, while 1 had respiratory tract infection after non-fatal drowning. Table 4 displayed details of the 6 cases. Our study echoes with the findings of To et al. (2), which was also carried out in Hong Kong, that the majority of *Shewanella*-associated infections did not have documented seawater contact. The warm climate in Hong Kong is favorable for *Shewanella* species (2, 35). Residing near seas and seacoasts was also identified as a potential risk factor for *Shewanella* infections (16). Hong Kong is located in close proximity to the seacoast, with high seafood demand and consumption. A possible route of transmission would be contaminated seafood in the waters of Hong Kong, which is ingested raw or undercooked (2, 9). These may explain the relatively large number of local cases recorded in Hong Kong.

### Clinical Presentations

*Shewanella*-associated infections are categorized into hepatobiliary infections, skin and soft tissue infections, bacteremia, and miscellaneous infections (5). Otitis media is a separate category of *Shewanella*-associated infection primarily described in the pediatric population and runs a relatively benign clinical course (34). Rarer involvement includes endophthalmitis (36), infective endocarditis (37), pericarditis (13), cerebellar abscess (38) and splenic abscess (39) described only in isolated case reports.

Hepatobiliary infections were predominant (60.4%, 93 out of 154 specimens) in our case series. This was consistent with the study by To et al., which was also carried out in Hong Kong (2), where intra-abdominal foci were the most typical site from which *Shewanella* species were isolated. In contrast, most other studies rarely isolated *Shewanella* species from the hepatobiliary system or intra-abdominally. Our findings may be attributed to the fact that a heavy proportion of our cohort had pre-existing hepatobiliary disorder (63.3%), which predisposed them to hepatobiliary tract infections. 51.6% (48 out of 93 specimens) of these hepatobiliary infections were classified as asymptomatic “colonization” while 40.9% (38 out of 93 specimens) were classified as “probable” infections. All isolates classified as “probable” infections and “colonization” had heavy co-isolates of enteric organisms like *Escherichia coli, Klebsiella* species and *Enterococcus* species. Similar polymicrobial and asymptomatic colonization of the PTBD was also observed in the study by To et al. (2). In *To*'s study, none of the bile specimens was classified as “definite” infections (2). In our series, 7.5% (7 out of 93) of bile specimens were classified as “definite” infections, all of them being bacteremia. Our study showed that in patients with an underlying hepatobiliary disorder, asymptomatic colonization of the biliary tract and existing drains by *Shewanella* and other enteric organisms was prevalent. These colonizing organisms may evolve into genuine and severe infections like bacteremia in appropriate clinical settings, such as acute biliary obstruction.

The overall 30-day mortality in our series was 8.6%. When only “definite” infections were considered, the mortality rate was significantly increased to 28.6%. In the Hong Kong study by To et al. (2), the overall 30-day mortality was 20.6%, much higher than our series. Among those with “definite” infections, they reported a mortality rate of 28%, and it was very similar to ours. Interestingly, one South African case series of 16 cases of *Shewanella* bacteremia reported only 7% overall mortality (14), suggesting a relatively benign clinical course even in cases of bacteremia. In a Denmark study by Holt et al., which focused on *Shewanella*-associated ear infections, the overall mortality was 0% due to the benign nature of ear infections (34). Overall, the reported mortality rate of *Shewanella*-associated infections in existing literature varied from 0 to 16% (2, 3, 5, 14, 34).

### Antibiotic Susceptibility

Our study showed that the majority (>97%) of *Shewanella* species were susceptible to ceftazidime and gentamicin. This was consistent with a prior study in Hong Kong carried out 10 years ago. *Shewanella* was found to be uniformly susceptible (100%) toward ceftazidime and aminoglycosides (gentamicin, amikacin, and tobramycin) (2). Susceptibility to cefoperazone-sulbactam, ciprofloxacin and piperacillin in our cohort also remained similar to that reported in the prior Hong Kong study (2). Alarmingly, resistance toward carbapenems has risen significantly over the past 10 years. Imipenem-resistant strains were detected in 3.7% of isolates in that study (2), hence 96.3% of *Shewanella* species were still imipenem-susceptible. Our study detected imipenem-resistant strains in 23.4% of isolates, with only 76.6% of isolates remaining imipenem-susceptible. In addition, resistance toward carbapenem may develop during therapy in initially susceptible strains (40). Kim et al. described a case of *Shewanella algae* bacteremia showing initial susceptibility to imipenem (40). Despite treatment with imipenem, the patient developed an epidural abscess requiring surgical drainage. The isolate from the epidural abscess was resistant to imipenem, and cefepime was used instead for subsequent treatment. It was postulated that a carbapenem-hydrolyzing class D beta-lactamase might play a role in the emergence of carbapenem resistance in the course of treatment for *Shewanella algae* (40, 41). Whole genome sequencing of *Shewanella algae* isolates showed expression of a higher level of blaOXA-55-like transcription and beta-lactamase activity in carbapenem-resistant strains, as compared to carbapenem-susceptible isolates (42). *Shewanella xiamenensis* was reported to harbor blaOXA-48-like class D carbapenemase genes (43). *Shewanella oneidensis* was shown to carry blaOXA-54 genes that encoded the class D beta-lactamase OXA-54 and led to significant hydrolysis of imipenem (44). Presence of these blaOXA genes for carbapenem-hydrolyzing class D beta-lactamase in *Shewanella* species could explain for reduced carbapenem activity toward *Shewanella* species.

Of note, carbapenem was a popular choice as an empirical antibiotic (23.3%, 30 out of 128 patients) in the treatment of *Shewanella*-associated infection in our study. 16.6% (5 out of 30) of patients received carbapenem as an inappropriate empirical choice, as carbapenem-resistant strains were eventually detected in these patients. Hospital mortality differed between those who received carbapenems appropriately and inappropriately at 4 and 20% respectively, although the difference failed to reach a statistical significance (*p* = 0.3103). However, a major confounder to interpret this association is our limited sample size. Overall, carbapenem susceptibility appeared to be variable and inconsistent. The use of carbapenems in *Shewanella* infections may need to be mindfully re-evaluated in the future in light of increasing resistance toward carbapenems and propensity to develop resistance toward carbapenems during treatment.

### Limitations

Our study was a retrospective study, and information on exposure history was subject to recall bias. For instance, the number of cases with seawater exposure prior to *Shewanella* infection may be under-estimated as marine contact history could be easily missed by the inattentive and unaware patient.

Secondly, different versions of CLSI MIC breakpoints were being used in our study period because of a change in the method employed for susceptibility testing in the 10-year period. This may have an impact on interpretation of the increasing carbapenem resistance as previously discussed. However, we must also acknowledge that the susceptibility pattern to other classes of antibiotics did not differ significantly despite a change in the CLSI criteria being used. Although we did not apply a consistent system of CLSI interpretation throughout the study period, the significantly increasing trend of carbapenem resistance should not be undermined or ignored.

Thirdly, identification of *Shewanella* species was based on MALDI-TOF Mass Spectrometry in our study. This may be unreliable as only few representative species are present in the current database. Increasing number of novel species have been identified in the *Shewanella* genus. For instance, *Shewanella chilikensis* and *Shewanella carassii* are phylogenetically related to *Shewanella algae* and are also implicated in human infections (42, 45, 46). Identification of these species requires dedicated techniques such as whole genome sequencing (42). It is possible that some samples of *Shewanella algae* in our study were in fact these novel *Shewanella* species, which were being “mis-identified” as *Shewanella algae*.

Fourthly, phylogenetic analysis or molecular characterization of antimicrobial resistance mechanisms were not carried out in our study.

## Conclusions

To our knowledge, this is the largest case series of *Shewanella* infections. Underlying hepatobiliary diseases and malignancies were consistent predisposing factors to *Shewanella* infections across the literature. In our cohort, hepatobiliary infections were predominant, and the majority were related to gallstone or bile duct stone disease. Patients with chronic skin ulcers, with or without marine exposure, were prone to *Shewanella*-associated skin and soft tissue infections. Most *Shewanella* species remained susceptible to third and fourth generation cephalosporins, as well as aminoglycosides. A trend of increasing carbapenem resistance was observed. The emergence of carbapenem resistance may also occur during treatment for an initially susceptible strain. Options other than carbapenems may need to be considered in treating *Shewanella*-associated infections.

## Data Availability Statement

The original contributions presented in the study are included in the article/supplementary material, further inquiries can be directed to the corresponding author/s.

## Ethics Statement

The studies involving human participants were reviewed and approved by Hong Kong East Cluster Ethics Committee of the Hospital Authority (HKECREC-2020-072). Written informed consent for participation was not required for this study in accordance with the national legislation and the institutional requirements.

## Author Contributions

WN helped with manuscript conception, data collection, data interpretation, writing of the manuscript, and critical review of the manuscript. H-PS helped with data collection, data interpretation, and critical review of the manuscript. KT and SS helped with critical review of the manuscript. All authors contributed to the article and approved the submitted version.

## Conflict of Interest

The authors declare that the research was conducted in the absence of any commercial or financial relationships that could be construed as a potential conflict of interest.

## Publisher's Note

All claims expressed in this article are solely those of the authors and do not necessarily represent those of their affiliated organizations, or those of the publisher, the editors and the reviewers. Any product that may be evaluated in this article, or claim that may be made by its manufacturer, is not guaranteed or endorsed by the publisher.
